# Satisfaction and Self-Confidence of Moroccan Nursing Students in Simulation-Based Learning and Their Associations with Simulation Design Characteristics and Educational Practices

**DOI:** 10.3390/nursrep15050138

**Published:** 2025-04-25

**Authors:** Hicham Blaak, Abdelmajid Lkoul, Hayat Iziki, Abdelhadi El Haddaouy, Ahmed Kharabch, Rachid Razine, Lahcen Belyamani, Majdouline Obtel

**Affiliations:** 1 Laboratory of Biostatistics, Clinical Research and Epidemiology, Department of Public Health, Faculty of Medicine and Pharmacy, University Mohammed V in Rabat, Rabat 10000, Morocco; abdelmajid_lkoul@um5.ac.ma (A.L.); kharbach.a.lbrce@gmail.com (A.K.); r.razine@um5s.net.ma (R.R.); m.obtel@um5r.ac.ma (M.O.); 2 High Institute of Nursing Professions and Technical Health, Agadir 80000, Morocco; hayat_iziki@um5.ac.ma; 3 Laboratory of Mother-Child Health and Nutrition Research, Faculty of Medicine and Pharmacy of Rabat, Mohammed V University of Rabat, Rabat 10000, Morocco; 4 Laboratory of Drugs Science, Faculty of Medicine and Pharmacy, Mohammed V University, Rabat 10000, Morocco; a.elhaddaouy@ispitsmeknes.ac.ma; 5 Laboratory of Cell Biology and Molecular Genetics, Department of Biology, Faculty of Sciences, Ibn Zohr University, Agadir 80000, Morocco; 6 Laboratory of Community Health, Preventive Medicine and Hygiene, Department of Public Health, Faculty of Medicine and Pharmacy, University Mohammed V in Rabat, Rabat 10000, Morocco; 7 Pedagogy and Research Unit of Public Health, Department of Public Health, Faculty of Medicine and Pharmacy, University Mohammed V in Rabat, Rabat 10000, Morocco; 8 Faculty of Medicine and Pharmacy, University Mohammed V in Rabat, Rabat 10000, Morocco; lbelyamani@um6ss.ma; 9 School of Medicine, University Mohammed VI of Health and Sciences, Mohammed VI Foundation of Health and Sciences, Casablanca 82403, Morocco

**Keywords:** simulation, satisfaction, self-confidence, educational practices, simulation design, nursing students, nursing education, standardized patient

## Abstract

**Background**: Nursing students must be able to enter clinical practice as safe, accurate, competent, and compassionate professionals. **Objectives:** The aim of this study was to investigate the effectiveness of simulation training on the satisfaction and self-confidence of undergraduate nursing students. **Methods:** A cross-sectional and correlational study was conducted among 151 students enrolled in the third semester of nursing. Data were collected using a questionnaire comprising three instruments: SSSCL, SDS, and EPQ. Pearson’s correlation analysis was used to examine the relationship between satisfaction and self-confidence. Multiple linear regression was conducted to assess the influence of simulation design and various educational practices on students’ satisfaction and self-confidence. **Results:** The results revealed high mean scores for satisfaction (4.41 ± 0.40) and self-confidence (4.50 ± 0.36). A moderate, significant positive correlation was found between self-confidence and satisfaction (r = 0.579, *p* < 0.001). Furthermore, various learning methods (B = 0.112, *p* = 0.037, 95% CI [0.007; 0.217]) and objectives/information clarity (B = 0.175, *p* = 0.040, 95% CI [0.008; 0.342]) had a significant positive effect on satisfaction. Similarly, active learning (B = 0.146, *p* = 0.020, 95% CI [0.023; 0.268]) and feedback (B = 0.154, *p* = 0.035, 95% CI [0.011; 0.297]) had a significant positive effect on self-confidence. **Conclusions:** This study confirms that simulation-based training effectively boosts nursing students’ satisfaction and self-confidence, supporting its integration as a key component of nursing education to better prepare them for clinical challenges.

## 1. Introduction

The training of healthcare professionals must incorporate innovative strategies adapted to digital natives, who are accustomed to digital tools and immersive environments. Among these strategies, simulation occupies a prominent place, offering students the opportunity to develop and refine their clinical skills in a controlled and safe environment [1]. Simulation is defined as an imitation of an object, a situation, or a real process used for skill practice, problem-solving, and clinical judgment [2]. It includes various modalities such as high- or low-fidelity mannequins, virtual reality, and standardized patients (SPs). The latter are individuals trained to realistically and reproducibly portray a patient in various clinical contexts [3]. In learning situations focused on psychosocial contexts requiring human interaction and emotional expression, the use of SPs is particularly valuable [4]. Body language, eye contact, spontaneous reactions, and other non-verbal cues provided by SPs enhance the authenticity of the learning experience [5]. These elements are difficult to replicate with mannequins, which lack emotional dynamism and natural interactivity. By providing a safe and realistic learning environment, simulation allows students to practice clinical procedures without risk to patients [6,7]. This gives them the opportunity to learn from their mistakes and progress at their own pace, thereby contributing to improved patient safety and quality of care [8].

Simulation not only enhances knowledge but also improves self-efficacy, critical thinking, problem-solving, and classroom immersion [9,10,11,12]. For example, simulation has been effectively used to improve students’ performance in managing postpartum hemorrhage [13], cardiac arrhythmia [14], medication administration [7,15], and surgical nursing [16]. It strengthens students’ ability to apply theory to practice, thereby fostering the development of decision-making [17] and clinical judgment skills [18,19]. Additionally, simulation enhances communication, teamwork [20,21,22,23], as well as self-confidence and learner satisfaction [4,24,25,26,27,28].

Satisfaction and self-confidence are considered key elements in evaluating the effectiveness of educational strategies. Satisfaction is defined as learners’ overall contentment and their positive perception of the learning experience, including the effectiveness of teaching methods, the adequacy of learning materials, and the enjoyment derived from the instructor’s teaching style [29]. It is associated with greater engagement and improved motivation [30], making it a key indicator of the effectiveness of educational interventions [31]. A satisfying learning experience fosters student engagement, enhances knowledge retention, and promotes a positive attitude toward the subject [29]. Learner satisfaction during simulation is essential for building their confidence and reflects a successful learning experience [32]. Self-confidence, on the other hand, refers to the belief that a person can accomplish a task or achieve a desired goal [33]. It is crucial for healthcare professionals to enable them to make informed decisions, communicate effectively, and perform clinical tasks with assurance [34]. Self-confidence supports more autonomous practice and contributes to the satisfaction of both caregivers and patients [35]. Conversely, a lack of confidence can lead to hesitation and compromise the quality of care [29]. The skills related to satisfaction and self-confidence acquired before graduation help students achieve lasting satisfaction in their professional careers [35].

In Morocco, simulation is gradually being implemented to address the shortage of clinical supervisors, the limited availability of clinical environments, and the challenges of accessing high-risk units. This still-emerging approach offers a vast field for empirical investigation. Therefore, the objective of this study was to examine the effectiveness of simulation using the National League for Nursing/Jeffries Simulation Framework (NLN/Jeffries Simulation Framework) on the satisfaction and self-confidence of undergraduate nursing students.

## 2. Materials and Methods

### 2.1. Study Design and Setting

**Figure 1:**
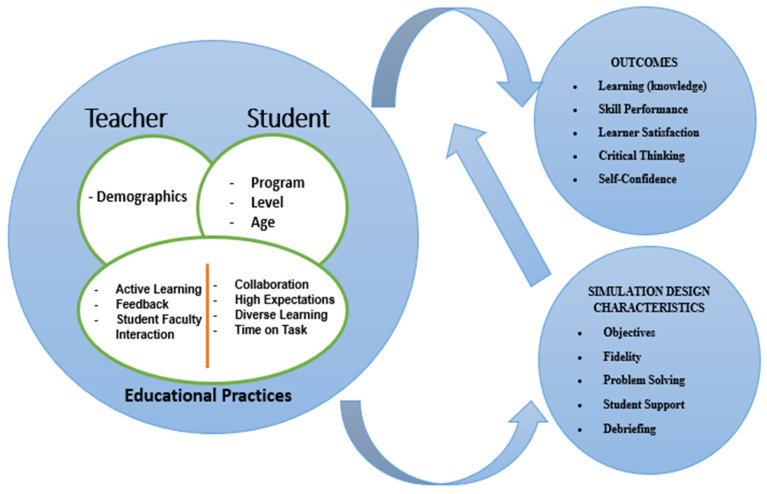
NLN/Jeffries Nursing Education Simulation Framework.

This study adopted a cross-sectional, descriptive, and correlational design. It was conducted at the Higher Institute of Nursing and Health Techniques (HINHT) in Agadir, Morocco. Established by Decree No. 2.13.658 of 30 September 2013, HINHT is a non-university higher education institution operating under the authority of the Ministry of Health of the Kingdom of Morocco [36]. The research aimed to examine the effect of simulation on nursing students’ satisfaction and self-confidence, based on the NLN/Jeffries Simulation Framework. This framework covers teacher, student, and educational practices, as well as simulation design, features, and outcomes. Simulation design and features include debriefing, student support, fidelity, problem solving, and clearly defined objectives. Educational practices comprise diversified learning, time on task, student–teacher interaction, active learning, high expectations, collaboration, and feedback. Finally, outcomes are assessed in terms of self-confidence, critical thinking skills, learner satisfaction, knowledge, and skill performance (Figure 1).

### 2.2. Study Population and Sampling

The study focused on nursing students enrolled at HINHT in Agadir, with an estimated population of 300 students. Recruitment was conducted through an announcement made during scheduled class sessions, in which the research team presented the study objectives, procedures, and ethical considerations. Students were informed that participation was entirely voluntary, and no academic or institutional consequences would result from declining or withdrawing from the study. A total of 187 students initially volunteered, but only 151 completed all stages of the research, forming the final sample. Inclusion criteria consisted of being enrolled in the third semester of the nursing program and voluntarily agreeing to participate. Students who had not completed the theoretical course or the required simulation scenarios, those enrolled in other levels of the nursing training program, or those who had not provided written informed consent were excluded. Prior to data collection, ethical approval was obtained from the ethics committee of Mohamed V University in Rabat, Morocco (No. 153/24), and all participants signed a written informed consent form in accordance with the Declaration of Helsinki.

### 2.3. Simulation Procedure

One week prior to the simulation activity, students received an in-depth theoretical course on the management of anaphylactic shock. At the end of the session, instructors presented the learning objectives without disclosing the full scenario, in order to preserve the authenticity and spontaneity of participants’ reactions. Learners were encouraged to review the course content and consult additional scientific references to enhance their preparation. To organize the simulation, students were divided into groups of five. A structured briefing session was held beforehand to reiterate the training objectives, outline the different phases of the scenario, and help students become familiar with the learning environment, available equipment, and resources. The scenario was based on the use of SPs—professionals specifically trained to portray, in a realistic and consistent manner, a patient experiencing an anaphylactic shock. These individuals underwent rigorous training supervised by simulation experts, enabling them to faithfully reproduce clinical signs, emotional reactions, and behaviors of a real patient. Prior to the session, SPs received a detailed briefing on their role, the scenario progression, and the educational objectives. They also followed a structured training protocol and participated in multiple rehearsals of the clinical case. These rehearsals, supervised by instructors, allowed for the refinement of performances to ensure consistency and standardization across simulations. To enhance realism and enable real-time monitoring of physiological parameters, SPs were supported by the open-source software Vital Sign Simulator (version 1.4.3, https://sourceforge.net/projects/vitalsignsim/, accessed on 20 April 2025), which allows dynamic control of hemodynamic parameters throughout the scenario.

The simulated scenario involved a 45-year-old patient admitted for consultation who had received an intravenous antibiotic injection to treat a bacterial infection. Minutes after the injection, the patient exhibited signs of anaphylactic shock: generalized skin rash, significant facial and lip swelling, breathing difficulties with stridor, dizziness, chest tightness, and severe hypotension (80/50 mmHg). The patient’s pulse was rapid (120 bpm) and thready. The primary objective for the students was to promptly recognize the clinical signs of anaphylactic shock, initiate appropriate emergency interventions, and ensure effective management. They were required to assess the patient’s vital signs, immediately stop the antibiotic administration, and administer an intramuscular adrenaline dose (0.3–0.5 mg) per protocol. Concurrently, they needed to deliver high-flow oxygen (15 L/min) via a high-concentration mask to improve oxygenation and continuously monitor the patient’s clinical status and vital signs. Students were also expected to prepare and, if necessary, administer complementary treatments such as antihistamines or corticosteroids, while maintaining effective communication with the emergency medical team. Finally, they had to accurately document interventions and their timing, as well as assess the effectiveness of actions taken to ensure optimal continuity of care during the patient’s transfer to an emergency team or hospital department.

At the end of the training, a one-hour debriefing session was conducted using Steinwachs’ three-phase model (descriptive, analytical, and application phases) [37] to deepen students’ understanding, enhance critical thinking, and promote the integration of learning into future clinical practice. During the descriptive phase, students recounted the sequence of events, shared emotional responses, and identified key challenges encountered during the simulation, fostering reflection on essential components of managing anaphylactic shock. The analytical phase involved a detailed examination of decisions and actions taken, allowing students to evaluate successes, errors, their rationale, and consequences, while drawing connections to real-world clinical scenarios. Finally, the application phase focused on developing actionable strategies to improve emergency responsiveness, team coordination, and protocol implementation in clinical settings.

Finally, all data collection instruments, including the Simulation Design Scale, the Educational Practices Questionnaire, and the Student Satisfaction and Self-Confidence in Learning Scale, were administered immediately after the conclusion of the debriefing session. This timing was deliberately chosen to ensure that students could reflect on the entire simulation experience (including preparation, participation, and debriefing) before responding, thereby enhancing the reliability and validity of their evaluations.

### 2.4. Data Collection Instruments

Data collection was carried out using a structured questionnaire, including socio-demographic data—namely age, gender, marital status, income, geographical origin, place of residence, semester validation status, and previous simulation experience—as well as three evaluation scales: the Simulation Design Scale (student version), the Pedagogical Practices Questionnaire (student version), and the Student Learning Satisfaction and Confidence Questionnaire. All three instruments used a five-point Likert scale to assess participants’ perceptions across the different dimensions. For each dimension, the mean score was calculated by dividing the sum of item responses by the number of items within the dimension. A score above 3.41 was considered high.

#### 2.4.1. The Simulation Design Scale (Student Version)

The Simulation Design Scale (SDS), student version, includes 20 items rated on a five-point Likert scale and is designed to evaluate essential elements of simulation-based learning aligned with adult education principles. The tool is structured into two sections: one assessing the perceived presence of specific features during the simulation, and the other evaluating how important these features are to the student. The scale encompasses five core dimensions. The first is clear objectives (five items), which assess whether the simulation goals were well defined and clearly communicated. The support dimension (four items) measures the extent of guidance and facilitation provided during the session. Problem-solving (five items) evaluates opportunities given to learners to independently address clinical problems based on their existing knowledge and skills. The feedback component (four items) assesses the quality and usefulness of feedback given to enhance learning. Lastly, fidelity or realism (two items) captures how closely the simulation mirrors real-life clinical settings. The reliability of this instrument was confirmed with a Cronbach’s alpha of 0.92 for the presence of features and 0.96 for their perceived importance [38].

#### 2.4.2. Educational Practices Questionnaire (EPQ) (Student Version)

The EPQ is a self-administered instrument composed of 16 items aimed at evaluating educational practices within simulation-based learning. It is structured into two sections: one assessing the presence of specific educational characteristics, and the other evaluating their perceived importance to learners. Each section encompasses four key dimensions: Active Learning (10 items), which explores the extent and value of learner engagement and opportunities for discussion; Collaboration (2 items), which considers the chance to interact and work with peers; Diverse Ways of Learning (2 items), which investigates the availability of varied instructional methods; and High Expectations (2 items), which ex-amines the clarity and communication of learning objectives and performance standards. The tool demonstrated good reliability, with Cronbach’s alpha coefficients of 0.86 for the presence of practices and 0.91 for their importance [39].

#### 2.4.3. Student Satisfaction and Self-Confidence in Learning (SSSCL)

The SSSCL is composed of 13 items distributed across two subscales. The satisfaction subscale includes five items measuring how content students were with the simulation experience. The self-confidence subscale includes eight items assessing the students’ confidence in applying what they learned. Responses are recorded on a five-point Likert scale ranging from Strongly Disagree to Strongly Agree. The reliability of the scale was established with a Cronbach’s alpha of 0.94 for the satisfaction subscale and 0.87 for the self-confidence subscale [40].

### 2.5. Data Analysis

Statistical analysis was conducted using descriptive tests, Pearson correlations, and multiple linear regressions. Descriptive statistics were used to characterize the sample (means, standard deviations, frequencies, and percentages). A Pearson correlation analysis was performed to examine the relationship between satisfaction and self-confidence. Finally, multiple linear regressions were conducted to identify factors influencing these dependent variables, assessing the impact of simulation design and various educational practices on student satisfaction and self-confidence. All analyses were performed using SPSS v27, with a significance threshold set at *p* < 0.05.

## 3. Results

### 3.1. Demographic Characteristics

The study sample consisted of 151 third-semester undergraduate nursing students, with a mean age of 20.01 ± 1.38 years. Women accounted for 64.2% of the sample, and nearly all participants were single (98%). The majority of students came from urban areas (73.5%) and had a middle-income level (71.5%), while 59.6% resided with their families. Academically, 98.0% of students had successfully completed their coursework, and 61.6% reported prior simulation experience (Table 1).

### 3.2. Satisfaction and Self-Confidence in Simulation-Based Learning

The majority of participants were highly satisfied overall (4.41 ± 0.40) with the simulation-based learning experiences. The highest satisfaction among students pertained to the teaching methods used in the simulations, with a strong score of (4.44 ± 0.73). Students also appreciated the way instructors conducted the simulations (4.44 ± 0.73) and found the instructional materials motivating and effective for their learning (4.44 ± 0.71). The lowest score in this category, though still high, was attributed to the variety of learning materials and activities (4.31 ± 0.79), which remains a strength but is slightly less pronounced compared to other aspects (Table 2). Additionally, most students perceived a significant increase in their self-confidence through the simulation experiences, with an overall score of (4.50 ± 0.36). The standout in this category was their belief in developing the skills and acquiring the knowledge necessary for clinical tasks (4.54 ± 0.72). Similarly, students acknowledged their personal responsibility in learning (4.54 ± 0.75), highlighting their commitment and autonomy. However, although scores were generally high, mastery of the content presented through simulation received a slightly lower score (4.44 ± 0.75), suggesting a potential need to strengthen this aspect to optimize learning. Finally, students reported knowing how to use simulations to acquire critical skills (4.51 ± 0.65) and how to seek help when needed (4.49 ± 0.66), demonstrating a good understanding of the tools and resources available to them (Table 2).

The correlation analysis between satisfaction and self-confidence showed a moderately significant association between these two variables (r = 0.579, *p* < 0.001) (Table 3).

A linear regression analysis was conducted to evaluate the influence of several variables (Active Learning, Collaboration, Diverse Ways of Learning, High Expectations, Objectives and Information, Support, Problem-Solving, Feedback, and Fidelity) on the dependent variables: Satisfaction and Self-Confidence.

### 3.3. Multivariate Regression of Satisfaction Based on Simulation Design and Educational Practices

The overall model explained 24.3% of the variance in this variable (R^2^ = 0.243; adjusted R^2^ = 19.5%), indicating a moderate contribution from the predictors included in the model. Specific learning methods (B = 0.112, *p* = 0.037, 95% CI [0.007; 0.217]) and goals/information (B = 0.175, *p* = 0.040, 95% CI [0.008; 0.342]) demonstrated significant positive effects on satisfaction. In contrast, other factors such as active learning (*p* = 0.058), collaboration (*p* = 0.352), and feedback (*p* = 0.567) showed no significant effects. The problem-solving variable exhibited a negligible and non-significant effect (B = 0.009, *p* = 0.918), suggesting no clear relationship with satisfaction in this model (Table 4).

### 3.4. Multivariate Regression of Self-Confidence Based on Simulation Design and Educational Practices

The overall model explained 26.7% of the variance in this variable (R^2^ = 0.267; adjusted R^2^ = 22.0%). Active learning (B = 0.146, *p* = 0.020, 95% CI [0.023; 0.268]) and feedback (B = 0.154, *p* = 0.035, 95% CI [0.011; 0.297]) had significant positive effects on self-confidence. Conversely, other variables, such as collaboration (*p* = 0.594), learning methods (*p* = 0.587), and support (*p* = 0.302), showed no significant effects. The problem-solving variable exhibited a non-significant negative effect (B = −0.106, *p* = 0.166) (Table 5).

## 4. Discussion

The results of this study highlight high satisfaction scores (4.41 ± 0.40) and self-confidence scores (4.50 ± 0.36), emphasizing the effectiveness of simulation as an innovative and immersive teaching method that strengthens these two essential dimensions of learning. These findings corroborate prior research highlighting the beneficial effects of simulation on learner satisfaction and self-confidence [41,42,43,44,45,46,47]. Comparing our results with those of previous studies, it appears that the observed satisfaction scores are higher than those reported in other contexts. For instance, a study conducted in Oman among 370 students reported an average satisfaction score of 3.97 [48]. Similarly, other studies involving nursing students have reported mean satisfaction scores of 4.38, 4.06, and 4.18, respectively [26,33,49]. Another study, conducted among medical students in critical care training, reported a mean satisfaction score of 4.20 [29]. This outcome underscores the effectiveness of the pedagogical strategies employed in our study, which not only align with students’ expectations but also appear to provide added value within the Moroccan educational context. It is worth noting that a cross-sectional study involving 305 participants, including healthcare professionals and students, reported an average satisfaction score of 4.72 [50]. These results can be explained by the immersive and realistic approach adopted in the design of the simulations. By recreating realistic clinical scenarios, simulation promotes active learning in a safe environment [51]. This learning approach enables students to develop and refine their skills while reflecting on their practices. The active engagement of learners in these simulated situations contributes to enhancing their satisfaction and self-confidence in managing clinical scenarios [52]. This relationship can be interpreted in light of social constructivism, which suggests that learning is built in interactive environments, where student interactions and active participation play a central role in skill acquisition [53].

A distinctive feature of our pedagogical approach is the use of SPs, which enhances engagement and realism in training scenarios, thereby reinforcing immersion in simulation. Unlike traditional methods that rely primarily on lectures and passive demonstrations, standardized patients allow students to interact with a responsive interlocutor adapted to the simulated clinical context. This immersive approach fosters the development of essential communication and clinical skills for nursing practice by providing immediate feedback on student performance and strengthening their ability to manage complex care situations [54]. Moreover, it enables more contextualized and emotionally engaging learning compared to high-fidelity mannequins. While mannequins are useful for simulating technical procedures, they cannot replicate the emotional nuances and human responses that standardized patients provide, facilitating better skill retention. Additionally, the use of standardized patients enhances the transferability of acquired skills to real-life situations, helping to bridge the gap between theory and practice [55]. Furthermore, the application of theoretical knowledge acquired during training in practical scenarios enriched by interaction with a standardized patient promotes problem-solving and clinical reasoning development [49]. By taking on collaborative roles and acting as team nurses, students adopt an active approach to knowledge acquisition while experimenting with different patient management strategies. This immersion also fosters peer learning, offering an exchange and reflection framework that stimulates creativity and adaptability. Thus, the fidelity and realism of simulation scenarios appear to have a positive effect on students’ ability to utilize their creativity, learn from one another, and apply their skills in a safe and controlled learning environment [42].

In terms of self-confidence, students expressed a high overall score of 4.50 ± 0.36, reflecting an enhanced sense of mastery and assurance in their clinical skills through simulation. This result stands out compared to previous studies, where reported scores generally ranged between 3.00 and 4.46 across various simulation contexts [33,49,50,52,56,57,58,59,60]. The highest scores were observed for two key aspects: students’ responsibility for learning what they need through simulation activities and the development of skills and acquisition of knowledge necessary for performing clinical tasks (4.54). These results emphasize the positive impact of simulation in fostering learner autonomy and preparing students for real clinical situations. Simulation-based training promotes individual learning in an interactive environment, where the student is at the center of the learning process, with the educator acting merely as a guide [61]. In traditional training methods, students typically take on a passive role as listeners, whereas in SP-based training, they actively engage in their learning. This active participation enhances performance and self-confidence. In the study by Dwood et al. [55], students indicated that standardized patient simulations were highly engaging experiences that increased their ability to interact with real psychiatric patients. Similarly, Godzik et al. [62] concluded that students established a high level of engagement with SPs, leading to a deeper level of understanding and learning, which was reflected in their feedback. Simulation-based training provides experiential learning, enhances students’ self-confidence, and develops their clinical decision-making skills. Additionally, it is important to note that simulation encourages a progressive assumption of responsibility, allowing students to practice, make mistakes, and learn from them in a controlled environment [63]. As a result, students gain confidence as they develop and acquire psychomotor skills, perform procedures, and provide patient care in critical or deteriorating scenarios [64]. Furthermore, simulation plays a crucial role in improving communication skills, both with patients and within the healthcare team [54]. It helps students organize their thoughts, establish priorities, and effectively communicate with other professionals. Thus, simulation contributes to the transition from student to independent clinical practice, reducing reliance on instructors, minimizing hesitation, and fostering greater student accountability in patient care [64].

Furthermore, our study revealed a moderate and significant positive correlation between satisfaction and self-confidence (r = 0.579, *p* < 0.001), highlighting the interdependence of these two dimensions. Similar results have been reported in the literature, suggesting that higher levels of satisfaction contribute to the development of self-confidence among students [29,56,65]. These findings reinforce the importance of providing engaging and satisfying learning experiences to promote the development of self-confidence among students. Student satisfaction is a key driver of their engagement in the learning process. When students are satisfied with the teaching methods used, they tend to participate more actively in the learning process [65]. Moreover, students who feel positively engaged in their education are more likely to develop confidence in their abilities. This increased self-confidence may lead to better decision-making and more effective skill execution, ultimately enhancing their clinical judgment and decision-making abilities [29,47].

Indeed, exploring the factors associated with student satisfaction and self-confidence is crucial for enhancing overall learning outcomes and developing effective simulation-based activities in nursing education. In this context, our study examined the relationship between simulation design characteristics, educational practices, and nursing students’ levels of confidence and satisfaction. Regarding educational practices, our findings highlighted that active learning and the diversification of teaching methods play a key role in improving student satisfaction and self-confidence. Although the effect of active learning on satisfaction was marginal (*p* = 0.058), its impact on self-confidence was found to be statistically significant (*p* = 0.020). These observations are consistent with previous studies [66]. Similarly, Olaussen et al. [52] demonstrated that simulation activities encourage students to engage fully, mobilizing their physical, psychological, intellectual, and interactional skills. Moreover, the variety of teaching methods used by instructors was positively correlated with student satisfaction. This correlation aligns with existing literature, which underscores the importance of adapting learning styles in simulations to foster critical thinking and reflexivity among students [51,67]. In this regard, educators should adopt diverse and flexible teaching strategies tailored to students’ individual preferences and cognitive profiles. For example, rotating roles (observer, active participant, team leader) during simulations can accommodate varying comfort levels and learning preferences. Pre-simulation briefings can incorporate elements that foster autonomous learning (e.g., guided research, self-assessment), while post-simulation debriefings should emphasize metacognitive strategies by encouraging students to analyze their performance and deepen their understanding through structured feedback.

In terms of collaboration, simulation serves as a pedagogical tool that promotes teamwork, allowing students to learn not only through their own actions but also through observation and peer interaction. While Gabbouj’s work highlights the positive influence of collaboration on satisfaction and self-confidence [66], our study did not identify a significant link between these variables. This result, consistent with some previous research [52], suggests that the impact of collaboration on these dimensions may vary depending on educational contexts or the specific implementation of simulation-based training.

Simulation design, as described in Jeffries’ simulation framework, is recognized as a key factor influencing the optimal experience of nursing students [68]. In this regard, our results confirm that the clarity of learning objectives and the relevance of information provided at the beginning of the simulation have a significant impact on student satisfaction. Establishing clear and well-defined educational objectives allows students to better prepare for the simulation and engage more actively in the learning process. This observation is supported by existing literature, which emphasizes that a clear and structured briefing fosters student engagement and contributes to a more positive perception of the learning experience [30,48,52].

Furthermore, repeated simulations have been shown to significantly improve content mastery by enabling learners to consolidate their knowledge through iterative practice. Repetition helps reduce cognitive load and anxiety, while enhancing students’ knowledge, skills, self-efficacy, satisfaction, and self-confidence [69,70,71]. By participating in repeated simulations, students progressively refine their skills and acquire greater autonomy and confidence in applying theoretical knowledge to clinical practice. Consequently, integrating repeated simulation sessions throughout the curriculum can be an effective pedagogical approach for reinforcing content mastery.

Consistent with previous findings, this study also revealed a positive correlation between feedback and students’ self-confidence [48]. Debriefing sessions following simulations play a crucial role in providing immediate feedback and evaluation. This process involves a guided discussion during which learners reflect on their performance, identify areas for improvement, and receive constructive feedback from the instructor. Such reflection helps reinforce learning, strengthen best practices, and address knowledge or skill gaps [54]. Moreover, feedback plays a key role in reducing anxiety and improving students’ perception of self-efficacy. During debriefing sessions, the review of recorded student performances served as a platform for constructive feedback from both instructors and peers. Another study also indicated that deliberate practice and video debriefing are effective simulation-based methods for nursing skill acquisition and self-assessment [72]. The active engagement of students during simulation and debriefing sessions also accommodates different learning styles, allowing students with diverse profiles to benefit from the experience [68]. For instance, visual learners can gain insights from practical demonstrations, while reflective learners appreciate in-depth discussions during debriefing. This pedagogical flexibility is essential to meet the needs of a diverse group. Furthermore, reflection facilitated by debriefing gives meaning to the simulation experience, providing the team with an opportunity to collaboratively analyze the event, think critically, and develop a plan for handling similar situations in the future [73].

According to Jeffries’ simulation theory, in a simulated scenario, learners are encouraged to identify a problem, develop solutions, and prioritize steps in their nursing care process to establish appropriate patient care objectives while exploring all possible options. However, in the present study, no statistically significant correlation was observed between problem-solving and students’ satisfaction or self-confidence. These findings are consistent with those reported in previous studies [66]. Nevertheless, a positive correlation between problem-solving and student satisfaction has been documented in several international studies that share similar objectives with our research, particularly in Norway, Oman, and Singapore [48,52,74]. Regarding support, our results did not reveal any significant correlation with satisfaction or self-confidence, which contrasts with previous studies that established a link between these variables [48,52]. Guinea et al. emphasized that the presence of adequate support and sufficient interactions between students and instructors during simulation enhanced students’ self-confidence in performing clinical activities [75]. As for simulation realism, this study did not identify a significant effect on satisfaction or self-confidence. However, Khasawneh et al. reported that simulation fidelity was positively correlated with satisfaction and self-confidence in simulation-based learning [48]. Although realism is a key aspect of simulation, its impact depends on students’ subjective perceptions, influenced by their clinical experience and expectations. In our study, participants may have given more importance to more engaging elements, such as interactivity or the clarity of learning objectives. Moreover, the use of standardized patients, while emotionally rich, may not have been perceived as realistic from a technical standpoint (e.g., automated hemodynamic responses, simulated medical devices), which could explain the neutral score observed. Furthermore, it is important to consider a methodological limitation of our measurement tool: the fidelity component of the SDS scale includes only two items, which may limit its sensitivity in detecting subtle effects. Other tools or qualitative approaches could be considered in future research to further explore the role of realism in simulation.

Although some variables did not show significant associations, our results highlight the importance of optimally leveraging simulation design characteristics and educational practices to improve academic performance. The positive correlations observed, in line with existing literature, support an integrated approach that considers both pedagogical practices and simulation design to optimize learning outcomes. It is therefore essential that simulation educators mobilize all available resources when planning activities to maximize their impact on educational outcomes. These findings further strengthen the case for systematically integrating simulation into nursing education programs, particularly for novice learners, thereby contributing to the improvement of the Moroccan educational system. Additionally, the data obtained could help convince nursing educators of the value of simulation as an essential teaching tool, capable of reducing errors in clinical practice and consequently improving patient care quality. Strengthening simulation-based learning practices would promote the training of highly skilled professionals by developing students’ competencies, knowledge, critical thinking, and clinical judgment. Finally, policymakers and educational leaders must ensure that nursing educators are proficient in the design, implementation, and evaluation of simulation activities. In this regard, the provision of comprehensive and effective training programs, covering various specialties, is essential to guarantee the achievement of the desired learning outcomes.

### Limitations and Future Directions

This study presents certain limitations that should be considered when interpreting the results. First, the use of a cross-sectional design does not allow for establishing causal relationships due to the lack of temporal follow-up. A longitudinal approach would be more appropriate to better understand the long-term effects of simulation-based learning. Additionally, the study was conducted in a single institution, limiting the generalizability of the findings and their applicability to other educational contexts or student populations. Moreover, the use of a convenience sample for participant recruitment introduces a selection bias, reducing the extent to which conclusions can be applied to a broader and more diverse population. Furthermore, this research focused exclusively on student satisfaction and self-confidence as indicators of learning, without considering other essential dimensions such as technical skills, knowledge acquisition, motivation, critical thinking, or real-world behavior. A more comprehensive evaluation of the impact of simulation would require integrating these aspects.

Another limitation is the absence of a baseline measure of self-confidence prior to the intervention. Without a pretest, it is difficult to determine whether the observed levels of self-confidence truly reflect an increase attributable to the simulation-based experience.

In addition, while the use of SPs offers numerous pedagogical benefits, this approach also presents practical limitations and barriers. SPs require extensive preparation, continuous training, and careful logistical coordination, which may lead to significant financial and organizational constraints for educational institutions. These constraints must be taken into account when planning the systematic integration of SP-based simulations into nursing education curricula.

For future research, it would be relevant to replicate this study with a larger sample and adopt probability sampling to enhance the generalizability of the results. Additionally, exploring other dimensions of learning, such as academic performance, critical thinking ability, motivation, and prior knowledge, while considering different levels of training, would provide a more comprehensive understanding of how simulation influences skill and knowledge development throughout nursing students’ academic journey. Expanding the scope of investigation in this way would help optimize educational practices and enhance the effectiveness of simulation in nursing education.

## 5. Conclusions

This study highlights the positive impact of simulation on nursing students’ satisfaction and self-confidence. The high overall mean scores and the strong correlation between satisfaction and self-confidence underscore the effectiveness of the implemented training program. Additionally, different learning modalities and the definition of educational objectives were positively correlated with satisfaction, while active learning and feedback were significantly correlated with self-confidence. These findings suggest that special attention should be given to simulation design characteristics and key elements of educational practices, as they serve as essential tools for optimizing simulation-based learning.

In conclusion, these results provide valuable insights for policymakers and educators, serving as a solid foundation for the development and implementation of training programs. They help bridge the gap between theory and practice in nursing education while facilitating students’ transition into their professional careers. These conclusions could also guide future research on enhancing simulation-based teaching methods, further strengthening the effectiveness of these training approaches.

## Figures and Tables

**Table 1:** Demographic characteristics of the participants.

Variables		N	%
Age (mean ± SD) 20.01 ± 1.38			
Gender	Female	97	64.20
	Male	54	35.80
Marital Status	Single	148	98.00
	Married	3	2.00
Income level	High	13	8.60
	Low	30	19.90
	Medium	108	71.50
Origin	Rural	40	26.50
	Urban	111	73.50
Residence type	Family	90	59.60
	Shared housing with friends	49	32.50
	University campus	12	7.90
Semester validation status	No	3	2.00
	Yes	148	98.00
Prior simulation training	No	58	38.40
	Yes	93	61.60

**Table 2:** Participants’ satisfaction and self-confidence scores.

	Mean	SD
Satisfaction with current learning		
The teaching methods used in this simulation were helpful and effective.	4.44	0.73
2. The simulation provided me with a variety of learning materials and activities to promote my learning the medical surgical curriculum.	4.31	0.79
3. I enjoyed how my instructor taught the simulation.	4.44	0.73
4. The teaching materials used in this simulation were motivating and helped me to learn	4.44	0.71
5. The way my instructor(s) taught the simulation was suitable to the way I learn.	4.43	0.71
**Overall Satisfaction**	4.41	0.40
**Self-confidence in learning**		
6. I am confident that I am mastering the content of the simulation activity that my instructors presented to me	4.44	0.75
7. I am confident that this simulation covered critical content necessary for the mastery of medical surgical curriculum.	4.44	0.74
8. I am confident that I am developing the skills and obtaining the required knowledge from this simulation to perform necessary tasks in a clinical setting.	4.54	0.72
9. My instructors used helpful resources to teach the simulation.	4.50	0.69
10. It is my responsibility as the student to learn what I need to know from this simulation activity.	4.54	0.75
11. I know how to get help when I do not understand the concepts covered in the simulation	4.49	0.66
12. I know how to use simulation activities to learn critical aspects of these skills	4.51	0.65
13. It is the instructor’s responsibility to tell me what I need to learn of the simulation activity content during class time	4.49	0.76
**Overall Self-confidence**	4.50	0.36

**Table 3:** Correlation between participants’ satisfaction and self-confidence scores.

Satisfaction	Self-Confidence	Pearson Correlation	*p*
4.41 (0.40)	4.50 (0.36)	0.579	<0.001

**Table 4:** Multivariate regression of satisfaction based on simulation design and educational practices (N = 151).

	Unstandardized Coefficients		Standardized Coefficients	t	*p*	95.0% Confidence Interval for B	
	B	SE	Beta			Lower Bound	Upper Bound
**Educational practices**							
Active learning	0.134	0.070	0.188	1.914	0.058	−0.004	0.272
Collaboration	−0.027	0.028	−0.074	−0.935	0.352	−0.083	0.030
Diverse ways of learning	0.112	0.053	0.196	2.111	0.037	0.007	0.217
High expectations	−0.056	0.062	−0.091	−0.902	0.369	−0.180	0.067
**Simulation design**							
Objectives and information	0.175	0.085	0.270	2.074	0.040	0.008	0.342
Support	0.037	0.058	0.070	0.641	0.522	−0.077	0.152
Problem solving	0.009	0.086	0.014	0.103	0.918	−0.161	0.179
Feedback	−0.047	0.082	−0.082	−0.574	0.567	−0.208	0.115
Fidelity	0.029	0.045	0.060	0.645	0.520	−0.060	0.119

**Table 5:** Multivariate regression of self-confidence based on simulation design and educational practices (N = 151).

	Unstandardized Coefficients		Standardized Coefficients	t	*p*	95.0% Confidence Interval for B	
	B	SE	Beta			Lower Bound	Upper Bound
**Educational practices**							
Active learning	0.146	0.062	0.228	2.356	0.020	0.023	0.268
Collaboration	0.013	0.025	0.042	0.534	0.594	−0.036	0.063
Diverse ways of learning	0.026	0.047	0.050	0.544	0.587	−0.067	0.119
High expectations	0.004	0.055	0.007	0.065	0.948	−0.106	0.113
**Simulation design**							
Objectives and information	0.062	0.075	0.105	0.822	0.413	−0.086	0.209
Support	0.053	0.051	0.111	1.035	0.302	−0.048	0.154
Problem solving	−0.106	0.076	−0.189	−1.394	0.166	−0.256	0.044
Feedback	0.154	0.072	0.298	2.130	0.035	0.011	0.297
Fidelity	0.000	0.040	0.000	−0.004	0.997	−0.079	0.079

## Data Availability

All data generated or analyzed during this study are included in this published article.

## Public Involvement Statement

No public involvement in any aspect of this research.

## Guidelines and Standards Statement

This manuscript was drafted in accordance with STROBE for cross-sectional studies.

## Abbreviations

The following abbreviations are used in this manuscript:

EPQ	Educational Practices Questionnaire
HINHT	Higher Institute of Nursing and Health Techniques
SDS	Simulation Design Scale
SPs	Standardized Patients
SSSCL	Student Satisfaction and Self-Confidence in Learning
